# Magnetic and Luminescence Properties of 8-Coordinate Holmium(III) Complexes Containing 4,4,4-Trifluoro-1-Phenyl- and 1-(Naphthalen-2-yl)-1,3-Butanedionates

**DOI:** 10.3390/molecules27031129

**Published:** 2022-02-08

**Authors:** Franz A. Mautner, Florian Bierbaumer, Ramon Vicente, Saskia Speed, Ánnia Tubau, Mercè Font-Bardía, Roland C. Fischer, Salah S. Massoud

**Affiliations:** 1 Institute of Physical and Theoretical Chemistry, Graz University of Technology, Stremayrgasse 9, A-8010 Graz, Austria; bierbaumerflorian97@gmail.com; 2 Departament de Química Inorgànica i Orgànica, Universitat de Barcelona, Martí i Franquès 1-11, E-08028 Barcelona, Spain; rvicente@ub.edu (R.V.); saskia.speed@qi.ub.es (S.S.); anniatubau@ub.edu (Á.T.); 3 Departament de Mineralogia, Cristallografia i Dipòsits Minerals and Unitat de Difracció de R-X, Centre Científic i Tecnològic de la Universitat de Barcelona (CCiTUB), Universitat de Barcelona, Solé i Sabarís 1–3, 08028 Barcelona, Spain; mercefont@ub.edu; 4 Institute of Inorganic Chemistry, Graz University of Technology, Stremayrgasse 9, A-8010 Graz, Austria; roland.fischer@tugraz.at; 5 Department of Chemistry, University of Louisiana at Lafayette, P.O. Box 43700, Lafayette, LA 70504, USA; 6 Department of Chemistry, Faculty of Sciences, Alexandria University, Moharam Bey, Alexandria 21511, Egypt

**Keywords:** lanthanides, holmium, X-ray, diketones, magnetic properties, luminescence

## Abstract

A new series of mononuclear Ho^3+^ complexes derived from the β-diketonate anions: 4,4,4-trifluoro-1-phenyl-1,3-butanedioneate (btfa^−^) and 4,4,4-trifuoro-1-(naphthalen-2-yl)-1,3-butanedionate (ntfa^−^) have been synthesized, [Ho(btfa)_3_(H_2_O)_2_] (**1a**), [Ho(ntfa)_3_(MeOH)_2_] (**1b**), (**1**), [Ho(btfa)_3_(phen)] (**2**), [Ho(btfa)_3_(bipy)] (**3**), [Ho(btfa)_3_(di-^t^bubipy)] (**4**), [Ho(ntfa)_3_(Me_2_bipy)] (**5**), and [Ho(ntfa)_3_(bipy)] (**6**), where phen is 1,10-phenantroline, bipy is 2,2′-bipyridyl, di-*^t^*bubipy is 4,4′-di-*tert*-butyl-2,2′-bipyridyl, and Me_2_bipy is 4,4′-dimethyl-2,2′-bipyridyl. These compounds have been characterized by elemental microanalysis and infrared spectroscopy as well as single-crystal X-ray difraction for **2**–**6**. The central Ho^3+^ ions in these compounds display coordination number 8. The luminescence-emission properties of the pyridyl adducts **2**–**6** display a strong characteristic band in the visible region at 661 nm and a series of bands in the NIR region (excitation wavelengths (λ_ex_) of 367 nm for **2**–**4** and 380 nm for **5** and **6**). The magnetic properties of the complexes revealed magnetically uncoupled Ho^3+^ compounds with no field-induced, single-molecule magnet (SMMs).

## 1. Introduction

The luminescent emissions of lanthanides in general, and specifically holmium complexes, have been known for decades, as they play crucial roles in research and have a wide range of useful applications [1,2,3,4,5,6,7,8,9,10,11,12,13,14,15,16,17,18,19,20,21,22,23,24,25,26,27,28]. Compared to other lanthanides, holmium was proved to serve as a good candidate to make quantum computers, where one bit of data can be stored on a single holmium atom set on a bed of magnesium oxide [23,24]. In addition, Ho is used to generate the strongest artificial magnetic fields when placed within high-strength magnets [25]; Ho-dopped yttrium iron garnet is used in optical insulators, microwave equipment, and in solid-state lasers [26], and is one of the colorant’s sources for yellow and red colors in glass and cubic zirconia [27].

Lanthanide ions, Ln^3+^ and their complexes, are known to exhibit narrow and characteristic *f*–*f* transitions of luminescent emissions that span from ultraviolet (UV) to visible and near-infrared (NIR) regions [1,2,3,29,30,31,32]. The *f–f* transitions in Ln^3+^ complexes are weak, but this process is enhanced via effective energy transfer from ligands or linker electrons to the central metal ions “antenna effect”, from which the emission occurs [1,2,3,20,21,32,33,34,35]. Most of the investigated complexes, such as Eu^3+^ and Tb^3+^, emit red or green luminescent light, respectively [36,37,38,39], but other Ln^3+^ complexes, such as those containing Yb^3+,^ Nd^3+^, and Pr^3+^ metal ions, exhibit luminescence in the near-IR region [40,41,42,43].

The lanthanide cations (Ln^3+^) as hard Lewis acids exhibit a strong binding affinity for *O*-donor ligands such as β-diketone compounds (HL) [43,44,45,46,47,48,49]. Typically electrically neutral tris complexes, Ln(L)_3_ are most likely to be formed [40,41,42,43,44,45,46,47,48,49,50,51,52,53,54,55], but in some cases, the anionic tetrakis complexes, (Cat^+^)[Ln(L)_4_]^−^ are also formed [49,56,57,58,59]. The two categories of these compounds exhibit good luminescent properties [40,41,42,43,44,45,46,47,48,49,50,51,52,53,54,55,56,57,58,59]. The luminescence efficiency of the β-diketonato complexes can be enhanced by the appropriate choice of the substituents on the β-diketone ligand because, in this way, the ligands’ triplet levels can be tuned to provide efficient energy transfer between the diketonato ligand and the lanthanide ion [60,61,62,63]. This has been observed when aromatic and fluorinated alkyl groups are incorporated into the β-diketone skeletons. This helps in reducing the nonradiative quenching of lanthanide luminescence [40,41,42,50,51,52,53,54,55,56,57,58,59,60,61,62,63]. In the anionic (Cat^+^)[Ln(L)_4_] complexes, additional tuning of the photophysical properties is possible by changing the counterion, Cat^+^, which in turn changes the structure of the complex and, in particular, the local coordination geometry of the metal ion [56,57,58,59].

**Scheme 1:**
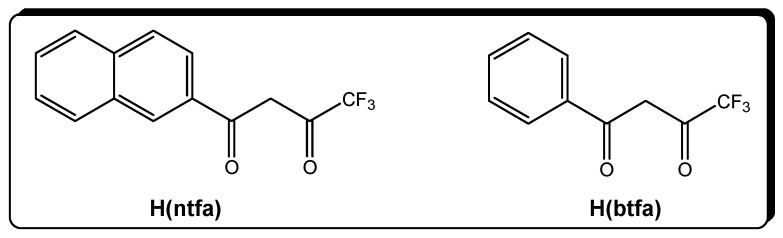
Structures of the β-diketones used in this study.

The rare-earth complexes with fluorinated-β-diketones (HL), such as L = 4,4,4-trifluoro-1-phenyl-1,3-butanedionate (btfa) and 4,4,4-trifuoro-1-(naphthalen-2-yl)-butane-1,3-dionate (L = ntfa) anions, have been extensively investigated. The structure formulas of H(btfa) and H(ntfa) are shown in Scheme 1. Among the Ln(III)-btfa complexes, half of them are for Eu(III) compounds [52,53,54,55,56,57,59,62,63,64], whereas the rest are for Dy(III) [50,51,60], Er(III) [55,61], Tb(III) [62], and Gd(III) [56,63]. In addition, small numbers were reported for Sm(III) [58], Pr(III) [42], and Ho(III) [65]. No structural results were found for La(III), Ce(III), Nd(III), Yb(III), nor Lu(III). In case of Ln(III)-ntfa, less structures were reported compared to the corresponding Ln(III)-btfa compounds, where most were obtained with Eu(III) [38,39,40,41,55,56,60,61,62,63,64,65,66], Gd(III) [43,53,55,56,57,66], and Pr(III) [42,45,48,65], some with Dy(III) [50,60,66] and Er(III) [55,65], as well as Tb(III) [62,66]. To the best of our knowledge, few structures were characterized with La(III) [49], Nd(III) [43], Ho(III) [65], and Sm(III) [65], but no structures for Ce(III), Yb(III), nor Lu(III) were found.

As part of a long project to explore the coordination properties and the physicochemical properties of the less-studied Ln^3+^ ions with the β-diketones, Ho(btf) and Ho(ntfa), the following studies were undertaken and devoted for the interaction of these two compounds with Ho^3+^ ions in the presence of different polypyridyl ligands.

## 2. Materials and Methods

### 2.1. Materials and Physical Measurements

4,4,4-Trifluoro-1-(phenyl)butane-1,3-dione, 4,4,4-trifluoro-1-(naphthalen-2-yl)-butane-1,3-dione, 4,4′-di-*tert*-butyl-2,2′-bipyridine, 5,5′-dimethyl-2,2′-bipyridine, and 2,2′-bipyridine were purchased from TCI, and the other chemicals were of analytical grade quality. Infrared spectra of solid complexes were either recorded on a Bruker Alpha P (platinum-ATR-cap) spectrometer (Bruker AXS, Madison, WI, USA) or a Thermo Scientific Nicolet IS5 spectrophotometer. Elemental microanalyses were carried out with an Elementar Vario EN3 analyzer (Langenselbold, Germany) at the Serveis Científics i Tecnològics of the Universitat de Barcelona. PXRD patterns were recorded with a Bruker D8 Advance powder diffractometer (Cu-Kα radiation) (Bruker AXS, Madison, WI, USA).

Solid-state fluorescence spectra of compounds **2**–**6** were recorded on a Horiba Jobin Yvon SPEX Nanolog fluorescence spectrophotometer (Fluorolog-3 v3.2, HORIBA Jovin Yvon, Cedex, France) equipped with a three-slit, double-grating excitation and emission monochromator with dispersions of 2.1 nm/mm (1200 grooves/mm) at room temperature. The steady-state luminescence was excited by unpolarized light from a 450 W xenon CW lamp and detected at an angle of 22.5° for solid-state measurement by a red-sensitive Hamamatsu R928 photomultiplier tube. Near Infra-red (NIR) spectra were recorded at an angle of 22.5° using a liquid-nitrogen-cooled, solid indium/gallium/arsenic detector (900–1600 nm). The instrument was adjusted to obtain the highest background-to-noise ratio with a band pass of 2 for the visible and 10 for the NIR measurements. The sample was mounted between two quartz plates. Spectra were corrected for both the excitation source light intensity variation (lamp and grating) and the emission spectral response (detector and grating).

The magnetic susceptibility and magnetization measurements were performed with a Quantum Design MPMS-XL SQUID magnetometer at the Magnetic Measurements Unit of the University of Barcelona. Pascal’s constants were used to estimate the diamagnetic corrections, which were subtracted from the experimental susceptibilities to give the corrected molar magnetic susceptibilities.

### 2.2. Synthesis of the Complexes

#### 2.2.1. [Ho(btfa)_3_(H_2_O)_2_] (**1a**)

To a methanol solution (10 mL) containing NaOH (6 mmol, 0.240 g), Hbtfa was added in an amount of 6 mmol, 0.130 g, and HoCl_3_·6H_2_O was added in an amount of 2 mmol, 0.759 g. The solution was stirred for 1 h at room temperature, then 80 mL of deionized water was added to the reaction mixture and stirred overnight. The light pink precipitate, which was obtained, was filtrated and dried in a desiccator overnight (yield: 1.194 g, 71%), Anal. Calcd. for C_30_H_22_F_9_HoO_8_ (846.4 g/mol): C, 42.6; H, 2.6%. Found: C, 42.5; H, 2.7%. Selected IR bands (cm^−1^): 3658 (m), 3462 (br), 1609 (s), 1575 (s), 1527 (m), 1488 (m), 1464 (m), 1329 (s), 1283 (s), 1245 (m), 1182 (s), 1144 (s), 1071(m), 945 (m), 777 (m), 694 (m), 631(m), 580 (m).

#### 2.2.2. [Ho(ntfa)_3_(MeOH)_2_] (**1b**)

A methanolic solution (10 mL) of Ho(NO_3_)_3_ 5H_2_O (281 mg, 0.64 mmol) and a methanolic solution (20 mL) of 4,4,4-trifluoro-1-(2-naphthyl)-1,3-butanedione (515 mg, 1.93 mmol) with 1M NaOH (2.0 mL) were dissolved. After 20 min of stirring, the 4,4,4-trifluoro-1-(2-naphthyl)-1,3-butanedione solution was added to the Ho(NO_3_)_3_ 5H_2_O solution. After 3 h of stirring, 30 mL of deionized water was added to complete the reaction. The mixture was stirred for 12 h at ambient temperature and then filtered. The obtained white powder was re-crystallized from MeOH and dried at 60 °C for 30 min (yield: 509 mg, 81%). Characterization: Anal. Calcd. for: C_44_H_32_F_9_HoO_8_ (1018.62 g/mol): C, 51.9; H, 3.2%. Found: C, 51.8; H, 3.1%. Selected IR bands (ATR-IR, cm^−1^): 3448 (m, br), 1602 (s), 1594 (m), 1568 (m), 1529 (m), 1458 (w), 1356 (w), 1285 (s), 1251 (m), 1184 (s), 1124 (s), 1073 (w), 958 (w), 865 (w), 824 (w), 794 (s), 762 (w), 684 (w).

#### 2.2.3. [Ho(btfa)_3_(L)] (**2**: L = phen; **3**: L = bipy; **4**: L = di-^t^Bubipy)

A general method was used to prepare the complexes **2**–**4**. An ethanol solution (15 mL) containing bipyridyl derivatives (1 mmol, **2**: 0.180 g 1,10-phenanthroline; **3**: 0.156 g 2,2′-bipyridine; **4**: 0.846 g 4,4′-di-*tert*-butyl-2,2′-bipyridine) was added to another ethanol solution (15 mL) containing [Ho(btfa)_3_(H_2_O)_2_] (1 mmol, 0.846 g). The solution was stirred for 30 min and then left to stand at room temperature. Single light pink crystals suitable for X-ray diffraction were obtained within a week. These were collected by filtration and dried with air.

[Ho(btfa)_3_(phen)] (**2**) (yield: 38%). Characterization: Anal. Calcd. for C_42_H_26_F_9_HoN_2_O_6_ (990.58 g/mol): C, 50.9; H, 2.6; N, 2.8%. Found: C, 50.7; H, 2.5; N, 2.8%. Selected IR bands (cm^−1^): 1610 (s), 1574 (s), 1522 (s), 1483 (m), 1476 (m), 1319 (m), 1291 (s), 1246 (m), 1178 (s), 1134 (s) 1077 (m), 846 (m), 763 (s), 770 (s), 631 (m), 580 (m).

[Ho(btfa)_3_(bipy)] (**3**) (yield: 80%). Characterization: Anal. Calcd. for C_40_H_26_F_9_HoN_2_O_6_ (966.56 g/mol): C, 49.7; H, 2.7; N, 2.9%. Found: C, 49.7; H, 2.5; N, 2.8%. Selected IR bands (cm^−1^): 1606 (s), 1569 (s), 1533 (m), 1472 (m), 1320 (m), 1279 (s), 1242 (m), 1177 (s), 1122 (s), 1067 (m), 1016 (m), 947 (m), 758 (s), 688 (s), 624 (s).

[Ho(btfa)_3_(di-^t^bubipy)] (**4**) (yield: 23%). Characterization: Anal. Calcd. for C_48_H_42_F_9_HoN_2_O_6_ (1078.77 g/mol): C, 53.4; H, 3.9; N, 2.6%. Found: C, 53.3; H, 3.7; N, 2.7%. Selected IR bands (cm^−1^): 2971 (w), 1612 (s), 1576 (m), 1539 (m), 1479 (m), 1403 (w), 1321 (m), 129 (s), 1248 (m), 1181 (s), 1127 (s), 1075 (m), 1026 (w), 948 (w), 848 (w), 766 (s), 699 (s), 635 (s), 580 (s).

#### 2.2.4. [Ho(ntfa)_3_(5,5′-Me_2_bipy)] (**5**)

[Ho(ntfa)_3_(MeOH)_2_] (127 mg, 0.125 mmol) and 5,5′-Dimethyl-2,2′-dipyridyl (28 mg, 0.15 mmol) were dissolved in 30 mL ethanol/acetone (3:1). The solution was stirred for approximately for 2 h. The mixture was filtered, and the mother liquor was left in an open atmosphere. After two weeks, pink crystals of **5** were obtained from the mother liquor (yield: 43 mg, 30%). Characterization: Anal. Calcd. for: C_54_H_36_F_9_HoN_2_O_6_ (1144.78 g/mol): C, 56.7; H, 3.2; N, 2.4%. Found: C, 56.6; H, 3.1; N, 2.5%. Selected IR bands (ATR-IR, cm^−1^): 1738 (w), 1608 (s), 1590 (m), 1566 (m), 1526 (m), 1506 (m), 1476 (w), 1384 (w), 1353 (w), 1284 (s), 1217 (w), 1183 (m), 1131 (s), 1073 (w), 956 (m), 935 (w), 862 (w), 790 (s), 748 (m), 681 (m), 569 (m), 517 (w), 467 (m), 416 (w).

#### 2.2.5. [Ho(ntfa)_3_(bipy)] (**6**)

[Ho(ntfa)_3_(MeOH)_2_] (124 mg, 0.122 mmol) was dissolved in 15 mL ethanol/acetone (4:1). 2,2´-bipyridyl (28 mg, 0.18 mmol) was dissolved in 15 mL ethanol/acetone (4:1). The solutions were combined and stirred approximately for 2 h. The mixture was filtered, and the mother liquor was left in an open atmosphere. After ten days, light pink crystals of **6** were obtained from the mother liquor (yield: 37 mg, 29%). Characterization of solvent-free compound: Anal. Calcd. for: C_52_H_32_F_9_HoN_2_O_6_ (1116.73 g/mol): C, 55.9; H, 2.9; N, 2.5%. Found: C, 55.8; H, 2.8; N, 2.6%. Selected IR bands (ATR-IR, cm^−1^): 1610 (s), 1591 (m), 1568 (m), 1528 (m), 1507 (m), 1460 (m), 1437 (w), 1387 (w), 1354 (w), 1286 (s), 1188 (m), 1121 (s), 1075 (w), 958 (m), 865 (w), 790 (s), 760 (m), 682 (w), 568 (m), 518 (w), 470 (m), 414 (w).

### 2.3. X-Ray Crystal Structure Analysis

Single crystals of **2**–**4** were set up in air on a Bruker-AXS D8 VENTURE diffractometer with a CMOS detector of **5** and **6** on a Bruker-AXS APEX II diffractometer (Bruker-AXS; Madison, WI, USA). The crystallographic data and details of the refinement are listed in Table 1. All the structures were refined by the least-squares method. Intensities were collected with multilayer monochromated Mo-Kα radiation. Lorentz polarization and absorption corrections were made in all the samples [67,68]. The structures were solved by direct methods using the SHELXS-97 computer program and refined by full-matrix least-squares method using the SHELXL-2014 computer program [69,70]. The non-hydrogen atoms were located in successive difference Fourier syntheses and refined with anisotropic thermal parameters on *F*^2^. Isotropic temperature factors were assigned as 1.2 or 1.5 times the respective parent for hydrogen atoms. For **6**, a SQUEEZE treatment was used to eliminate disordered solvent molecules. Further programs used: Mercury [71] and PLATON [72]. CCDC 2120112-2120116 contains the supplementary crystallographic data for **2**–**6**, respectively.

## 3. Results and Discussion

### 3.1. Synthesis and IR Spectra of the Complexes

The precursor complexes [Ho(btfa)_3_(H_2_O)_2_] (**1a**) and [Ho(ntfa)_3_(MeOH)_2_] (**1b**) were prepared by the reaction of methanolic solutions containing Ho(III) salts, beta-dicetonate molecules (Hbtfa) or (Hntfa), respectively, and NaOH in the stoichiometric ratio 1:3:3, followed by stirring the resulting solution in H_2_O. The PXRD pattern confirmed that **1a** is isostructural with [La(btfa)_3_(H_2_O)_2_] [47] and **1b** is isostructural with [Pr(nfa)_3_(MeOH)_2_] [48]. The interaction of [Ho(btfa)_3_(H_2_O)_2_] with poly-pyridyl compounds phen, bipy, and di-^t^bubipy in EtOH afforded the light-pink crystalline adducts [Ho(btfa)_3_(phen)] (**2**), [Ho(btfa)_3_(bipy)] (**3**), and [Ho(btfa)_3_(di-^t^bubipy)] (**4**), respectively, whereas the interaction of [Ho(ntfa)_3_(MeOH)_2_] with poly-pyridyl compounds 5,5′-Me_2_bipy and bipy in ethanol/acetone mixtures afforded the crystalline adducts [Ho(ntfa)_3_(5,5′-Me_2_bipy)] (**5**) and [Ho(ntfa)_3_(bipy)] (**6**) with reasonable yields (38–80%). The approach used here for the synthesis of complexes **2**–**6** is similar to that successfully employed in similar Ln(III) (Ln = La, Pr, Nd) mono bipyridyl adducts [47,48,49]. The isolated complexes were structurally characterized by elemental microanalyses and by IR spectroscopy, as well as by single-crystal X-ray crystallography for **2**–**6**.

The IR spectra of complexes **2**–**6** display general characteristic features. The strong vibrational band observed over the frequency range 1605–1615 cm^−1^ is typically assigned to the coordinated carbonyl stretching frequency, ν(C=O) [47,48,49]. The broad band centered at 3462 cm^−1^ in **1a** and 3448 cm^−1^ in **1b** is assigned to the ν(O-H) stretching frequency of the coordinated aqua/methanol ligands.

### 3.2. Description of the Crystal Structures **2**–**6**

**Figure 1:**
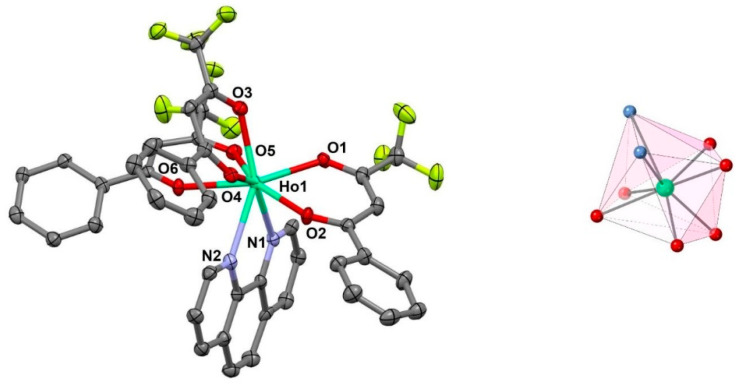
Left: partially labeled structure [Ho(btfa)_3_(phen)] (**2**). Color code: turquoise = Ho, red = O, yellow = F, grey = C. Right: coordination polyhedron of Ho(III) ion in compound **2**.

**Figure 2:**
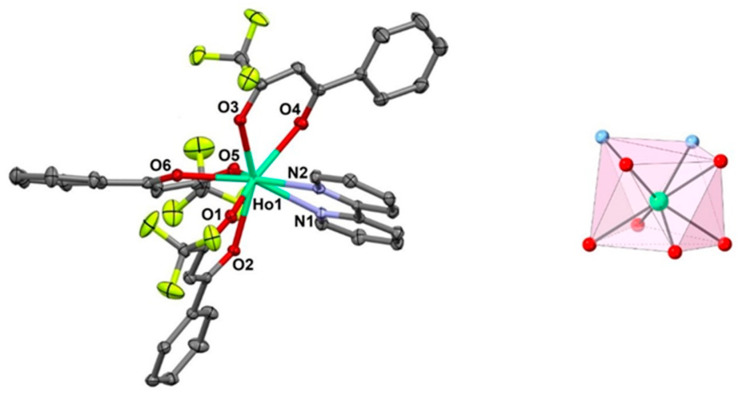
Left: partially labeled structure [Ho(btfa)_3_(bipy)] (**3**). Color code: turquoise = Ho, red = O, yellow = F, blue = N, grey = C. Right: coordination polyhedron of Ho(III) ion in compound **3**.

**Figure 3:**
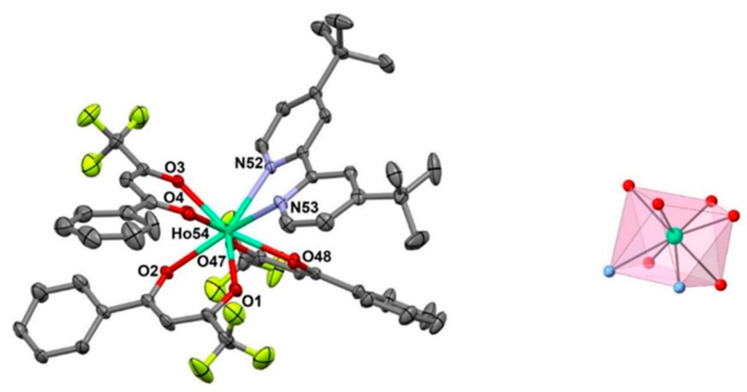
Left: partially labeled structure [Ho(btfa)_3_(di-^t^bubipy)] (**4**). Color code: turquoise = Ho, red = O, yellow = F, blue = N, grey = C. Right: coordination polyhedron of Ho(III) ion in compound **4**.

**Figure 4:**
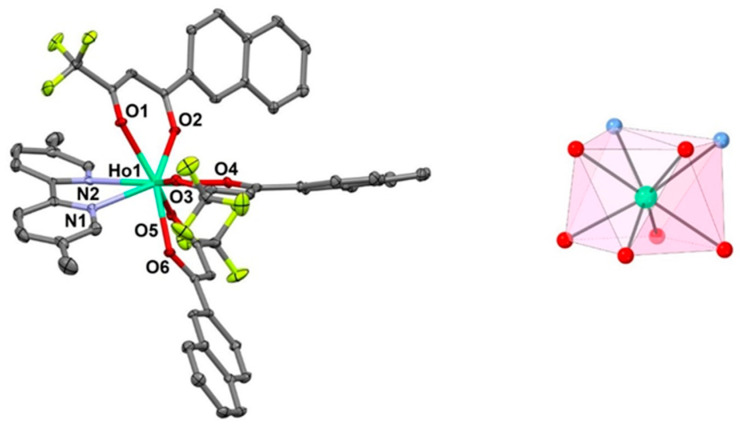
Left: partially labeled structure [Ho(ntfa)_3_(5,5′-Me_2_-bipy)] (**5**). Color code: turquoise = Ho, red = O, yellow = F, blue = N, grey = C. Right: coordination polyhedron of Ho(III) ion in compound **5**.

**Figure 5:**
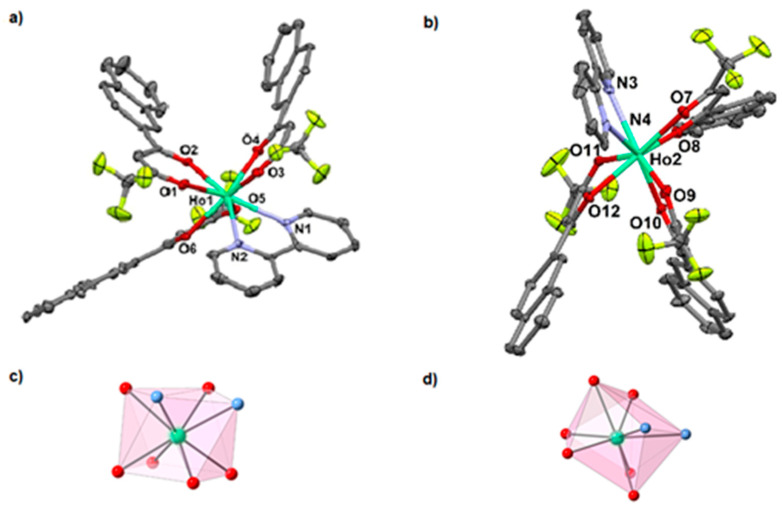
(**a**) Partially labeled structure **of** [Ho(ntfa)_3_(bipy)] (**6**): (**a**) Ho atom 1, (**b**) Ho atom 2, (**c**) coordination polyhedron of Ho atom1 and (**d**) coordination polyhedron of Ho atom 2. Color code: turquoise = Ho, red = O, yellow = F, blue = N, grey = C.

Molecular plots and coordination figures of **2**–**6** complexes are depicted in Figure 1, Figure 2, Figure 3, Figure 4 and Figure 5, and selected bond parameters are summarized in Table 2. Each Ho(III) center of the neutral and monomeric complex **2**–**6** are ligated by six oxygen donor atoms of three β-diketonato ligands anions (btfa^−^) for **2**–**4** or (ntfa^−^) for **5** and **6**, respectively, in the chelating coordination mode. The coordination number (CN) 8 in **2** is completed by two *N*-donor atoms of one phen chelating ligand. The Ho-N/O bond lengths in **2** are in the range of 2.3051(2)–2.5549(2) Å. The CN = 8 in **3**–**6** of the HoO_6_N_2_ coordination sphere around Ho is achieved by the ligation of one 2,2′-bipy (**3** and **6**), di-^t^Bu-bipy (**4**), and 5,5′-Me2-bipy (**5**) chelating ligands, respectively. The Ho-N/O bond lengths for **3** are in the range of 2.287(2)-2.527(3) Å, for **4**, from 2.292(3) to 2.541(4) Å, for **5**, from 2.293(4) to 2.518(4) Å, and for **6**, from 2.268(6) to 2.530(7) Å, respectively. The O-Ho-O bite angles of the β-diketonato groups fall in the range from 71.5(2) to 76.21(1)° in **2**–**6**, whereas the corresponding N-Ho-N bite angles of the chelating phen, bipy, di-^t^bu-bipy, and 5,5′-Me_2_bipy ligands in compounds **2**–**6** vary from 63.80(13) to 64.66(1)°.

Various non-covalent interactions (ring···ring, C-H(X)···ring [72], hydrogen bonds) are summarized in Tables S1–S5 for compounds **2**–**6**, respectively.

In order to analyze the degree of distortion of the coordination polyhedra for compounds **2**–**6** from their ideal polyhedron geometry, calculations using the continuous shape measures theory with the SHAPE software were performed [73,74]. The HoO_6_N_2_ coordination polyhedron of **2**–**6** shows an intermediate distortion between various ideal eight-vertex polyhedra geometries. These are a square antiprism (SPAR-8), triangular dodecahedron (TDD-8), and biaugmented trigonal prism (BTPR-8) with continuous shape values of 1.382, 1.236, and 1.779 for **2**; 0.553, 2.351, and 2.051 for **3**; 0.417, 2.515, and 2.254 for **4;** and 0.497, 2.210, and 1.958 for **5**.

The corresponding calculations of the degree of distortion of the HoO_6_N_2_ coordination polyhedra of compound **6** [(Ho(ntfa)_3_(bipy)] reveals an intermediate distortion between various coordination polyhedra geometries. These are a square antiprism (SPAR-8), triangular dodecahedron (TDD-8), and biaugmented trigonal prism (BTPR-8) with continuous shape measures values of 0.412, 2.348, and 2.33 for Ho(1)O_6_N_2_ and 0.3944, 2.165, and 2.115 for Ho(2)O_6_N_2_.

### 3.3. Photoluminescence of the Complexes

**Figure 6:**
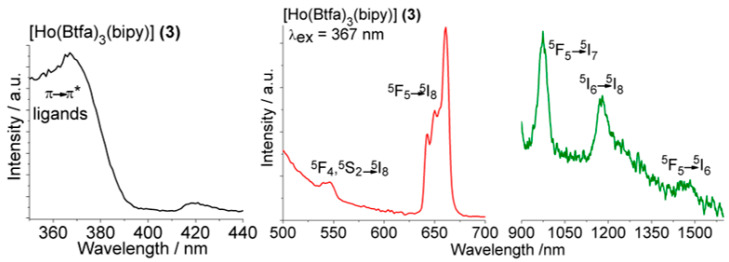
Spectra for complex **3**. Luminescence excitation (**black line**), emission in the visible range (**red line**), and in the NIR (**green line**) regions.

**Figure 7:**
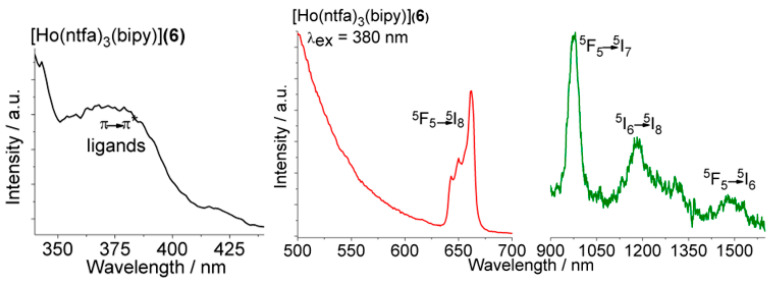
Spectra for complex **6**. Luminescence excitation (**black line**), emission in the visible (**red line**), and in the NIR (**green line**) regions.

The luminescence spectra of compounds **2**–**6** were measured in the solid state at room temperature. The excitation spectra recorded at the emission wavelength (λ_em_) of 661 nm reveal a broad, intense band around 367 nm for **2**–**4** and 380 nm for **5** and **6**. This broad band corresponds to the π → π* transition from the ligands. The luminescence emission spectra of the samples were recorded upon the excitation wavelengths (λ_ex_) of 367 nm for **2**–**4** and 380 nm for **5** and **6**. All spectra display a characteristic band at 661 nm (^5^F_5_ → ^5^I_8_) corresponding to the metal-centered emission and is assigned to the Ho^3+^ *f-f* transition from the ^5^F_5_ excited state to the ^5^I_8_ ground state. For this band, the Stark splitting of the degenerate 4*f* levels under the crystal field is perceived. In addition, compounds **2**–**4** showed a weak band at 545 nm, which can be assigned to an *f-f* transition from higher-energy states (^5^F_4_, ^5^S_2_) to the ground state ^5^I_8_ [75,76,77]. The triplet states of the ntfa and btfa ligands were calculated by Sato and Wadain Gd(III) complexes [78], taking into account the sensitization effect of the energy transfer from the singlet state of the ligand (S_1_) to the lower-in-energy ligand triplet state (T_1_) through the intersystem crossing. These calculations showed that the ntfa T_1_ state falls around 19,600 cm^−1^ for ntfa and 21,400 cm^−1^ for btfa. Thus, we can suggest that the energy transfer from the T_1_ of the ntfa ligand to the ^5^F_4_ and ^5^S_2_ (18,348 cm^−1^) thermal state is inefficient because the two states are too close in energy, and as a result, the ^5^F_4_, ^5^S_2_ → ^5^I_8_ transitions are not identified for compounds **5** and **6**, but they are seen for btfa complexes **2**–**4** [79]. Typical representative UV-Vis and luminescence emission spectra (Vis and NIR regions) are depicted in Figure 6 for **3** and Figure 7 for **6** as representatives of the two categories of **2**–**4** and **5** and **6** complexes, respectively (for luminescence spectra of **2**, **4**, and **5**, see Figures S12–S14).

Furthermore, the luminescence emissions of the compounds **2**–**6** were recorded in the NIR region from 900 to 1600 nm, where three weak bands were detected at 973, 1179, and 1474 nm. The first and most intense band is assigned to the ^5^F_5_ → ^5^I_7_ transition. The band located at 1179 nm accounts for the ^5^I_6_ → ^5^I_8_ transition; the very weak band at 1474 nm corresponds to the ^5^F_5_ → ^5^I_6_ transition [80]. The results obtained here agree with other Ho(III) coordination compounds, where the study of the sensitization of Ho^3+^ luminescence by the energy transfer from chromophore ligands has been performed [81,82,83,84,85].

### 3.4. Magnetic Properties of the Complexes

#### 3.4.1. Ac Magnetic Susceptibility Studies

In order to study the dynamic magnetic properties and the possible Single Molecular Magnet (SMM) behavior (slow relaxation of magnetization) of the synthesized compounds, ac magnetic susceptibility, measurements were recorded for solvent-free compounds **2**–**5**. Compounds **2**–**5** do not show a dependence on the in-phase and out-of-phase components in front of the temperature and frequency, neither in the minimum dc field (0 T) nor in the maximum applied dc magnetic field (0.1 T). Therefore, these compounds do not show slow relaxation of the magnetization and consequently will not show SMM’s behavior.

#### 3.4.2. Dc Magnetic Susceptibility Studies

**Figure 8:**
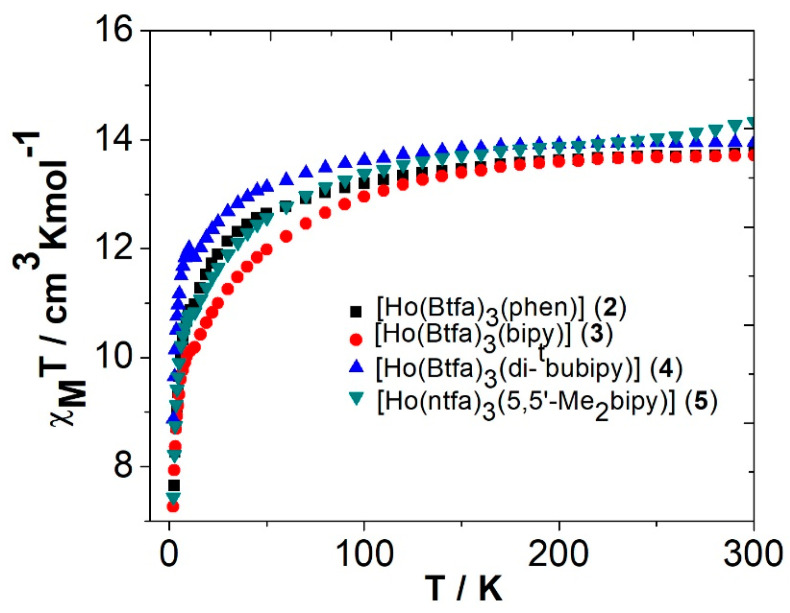
χ_M_T vs. T plots for compounds **2**–**5**.

**Figure 9:**
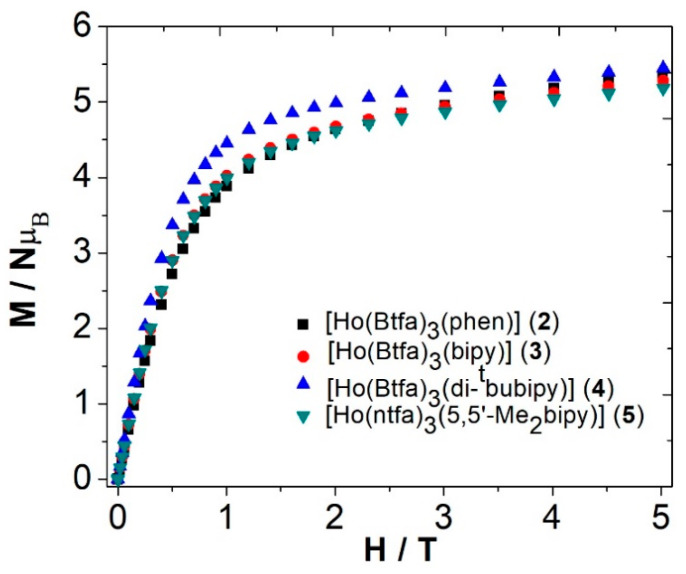
Field dependence of the magnetization plots at T = 2 K for compounds **2**–**5**.

Powder samples of complexes **2**–**5** were measured under applied magnetic fields of 0.3 T (300–2 K). The data are plotted as *χ*_M_*T* products versus *T* in Figure 8. Magnetization dependence of the applied field at 2 K for compounds **2**–**5** was also recorded and is shown in Figure 9.

The magnetic measurement on **2**–**5** reveals that the χ_M_T values at 300 K are 13.8, 13.7, 13.9, and 14.3 cm^3^ K mol^−1^, respectively, which are in the range of the theoretical value for a magnetically uncoupled Ho(III) compound (14.07 cm^3^·K·mol^−1^) in the ^5^I_8_ ground state (g_J_ = 5/4) [86]. On cooling the samples, χ_M_T values remain constant up to 125 K. Below this temperature, χ_M_T values decrease to finite values of 6.8, 7.3, 8.9, and 7.4 cm^3^·K·mol^−1^ at 2 K for compounds **2**–**5**, respectively. The decrease in χ_M_T values at low temperatures could be due to the depopulation of the sublevels generated for the spin–orbit coupling and the ligand-field effect (Stark sublevels).

Magnetization dependence on magnetic static applied field at *T* = 2 K for complexes **2**–**5** (Figure 9) reveals no saturation at high fields with similar values of 5.4, 5.3, 5.4, and 5.2 *Nµ*_B_ at 5 T for **2**–**5**, respectively. The magnetization saturation point expected for a mononuclear Ho^3+^ complex should be ≈4 N_A_µ_B._

**Figure 10:**
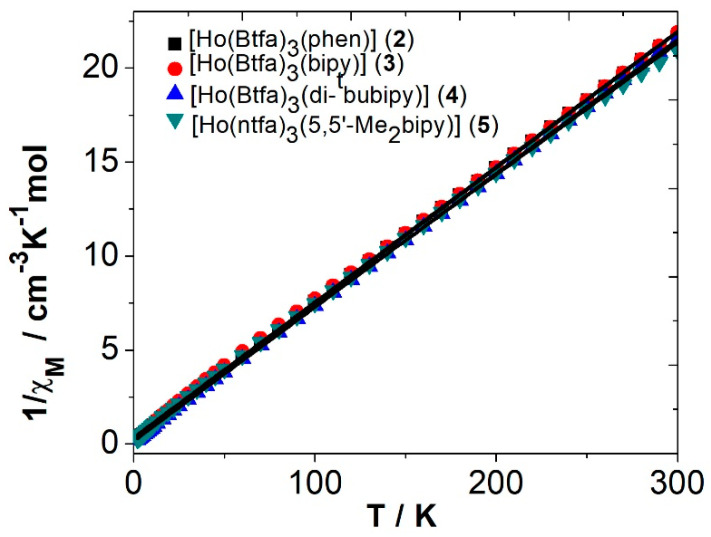
1/χ_M_ vs. T plots for compounds **2**–**5**. Solid lines represent the fitting using the Curie–Weiss law 1/χ_M_ = (T − θ)/C.

The 1/χ_M_ versus T plots for **2**–**5** are shown in Figure 10. Between 2 K and 300 K, the 1/χ_M_ versus T plots are linear for the four compounds and well described by the Curie–Weiss law 1/χ_M_ = (T–θ)/C, where C = 13.9 cm^3^·K·mol^−1^ and θ = −4.9 K for **2**, C = 13.9 cm^3^·K·mol^−1^ and θ = −3.50 K for **3**, C = 14.0 cm^3^·K·mol^−1^ and θ = −2.3 K for **4**, and C = 14.3 cm^3^·K·mol^−1^ and θ = −4.6 K for **5**.

## 4. Conclusions

A novel series of five mono-bipyridyl adducts of Ho^3+^-trifluoro-phenyl (btfa^−^) and -naphthalen-2-yl (ntfa^−^) β-diketonato complexes [Ho(btfa)_3_(phen)] (**2**), [Ho(btfa)_3_(bipy)] (**3**), [Ho(btfa)_3_(di-^t^bubipy)] (**4**), [Ho(ntfa)_3_(Me_2_bipy)] (**5**), and [Ho(ntfa)_3_(bipy)_2_] (**6**) were synthesized from their precursors diaqua tris(β-diketonato) species. The compounds were structurally characterized, where coordination numbers CN = 8 were observed. The distortion of the coordination polyhedra of Ho^3+^ centers was analyzed with the SHAPE program. All the complexes display CN 8. In a fashion that is similar to their Ln^3+^ analog complexes (Ln = La, Pr, and Nd) derived from the same set of ligands [47,48,49]. The solid-state luminescence emission of the complexes revealed a strong, intense emission band at 661 nm in the visible and three other bands in NIR regions. The magnetic measurements of the complexes **2**–**5** revealed that the χ_M_T values are within the range of 14.0 ± 0.3 cm^3^·mol^−1^·K at 300 K, which is predicted for a magnetically uncoupled Ho^3+^ compound (14.07 cm^3^·mol^−1^·K) in the ^5^I_8_ ground state (g_J_ = 5/4) [86]. The luminescence emission and magnetic results reported here for the Ho^3+^ compounds demonstrate that these properties are not significantly affected by either the small changes in the geometrical shape of the Ho^3+^ complexes or their local symmetry. Additionally, results are almost independent of the nature of the ancillary bipyridyl ligands or the nature of the β-diketone coligands. Similar results were obtained with pyridyl adducts derived from the same coligands with Pr(III) and Nd(III) compounds [48,49].

## Figures and Tables

**Table 1:** Crystal data and details of the structure determination of compounds **2**–**6**.

Compound	2	3	4	5	6
Empirical formula	C_42_H_26_F_9_HoN_2_O_6_	C_40_H_26_F_9_HoN_2_O_6_	C_48_H_42_F_9_HoN_2_O_6_	C_54_H_36_F_9_HoN_2_O_6_	C_52_H_32_F_9_HoN_2_O_6_
Formula mass	990.58	966.56	1078.76	1144.78	1116.73
System	Monoclinic	Monoclinic	Triclinic	Orthorhombic	Orthorhombic
Space group	P21/c	P21/n	P-1	Pca21	Pna21
a (Å)	9.6058(7)	11.0408(10)	12.3569(16)	20.2138(9)	20.7013(6)
b (Å)	36.627(2)	22.6440(18)	13.6076(18)	11.7503(5)	10.9059(3)
c (Å)	10.7464(7)	15.2463(13)	14.3853(18)	19.5852(7)	42.3027(10)
α (°)	90	90	92.478(5)	90	90
β (°)	92.932(3)	101.972(3)	99.883(5)	90	90
γ (°)	90	90	105.233(5)	90	90
V (Å3)	3776.0(4)	3728.8(6)	2289.3(5)	4651.8(3)	9550.5(4)
Z	4	4	2	4	8
μ (mm^−1^)	2.192	2.218	1.815	1.792	1.744
D_calc_ (Mg/m^3^)	1.742	1.722	1.565	1.635	1.553
θ max (°)	26.420	34.495	27.171	28.998	28.000
Data collected	91277	103169	50752	93718	264468
Unique refl./R_int_	7737/0.0836	15617/0.0776	10082/0.0390	12315/0.0812	23060/0.0515
Parameters/Restraints	542/0	523/0	601/0	651/1	1262/19
Goodness-of-fit on F^2^	1.120	1.050	1.131	1.012	1.165
R1/wR2 (all data)	0.0615/0.1342	0.0466/0.0809	0.0445/0.1042	0.0374/0.0632	0.0466/0.1060

**Table 2:** Selected bond distances (Å) and bite angles (°) for compounds **2**–**6**.

**Compound 2**		**Compound 3**		**Compound 4**	
Ho1-O1	2.3111(2)	Ho1-O1	2.315(2)	Ho54-O1	2.292(3)
Ho1-O2	2.3051(2)	Ho1-O2	2.343(2)	Ho54-O2	2.331(3)
Ho1-O3	2.3064(2)	Ho1-O3	2.297(2)	Ho54-O3	2.323(3)
Ho1-O4	2.3139(2)	Ho1-O4	2.326(2)	Ho54-O4	2.320(3)
Ho1-O5	2.3647(2)	Ho1-O5	2.287(2)	Ho54-O47	2.305(3)
Ho1-O6	2.3225(2)	Ho1-O6	2.330(2)	Ho54-O48	2.323(3)
Ho1-N1	2.5477(2)	Ho1-N1	2.524(2)	Ho54-N52	2.535(4)
Ho1-N2	2.5549(2)	Ho1-N2	2.527(3)	Ho54-N53	2.541(4)
O1-Ho1-O2	72.59(1)	O1-Ho1-O2	72.58(7)	O1-Ho54-O2	73.50(11)
O3-Ho1-O5	72.74(1)	O3-Ho1-O4	72.69(3)	O3-Ho54-O4	73.70(13)
O4-Ho1-O6	76.21(1)	O5-Ho1-O6	73.61(8)	O47-Ho54-O48	72.77(11)
N1-Ho1-N2	64.66(1)	N1-Ho1-N2	63.89(8)	N52-Ho54-N53	63.80(13)
**Compound 5**		**Compound 6**			
Ho1-O1	2.336(4)	Ho1-O1	2.273(6)	Ho2-O7	2.342(6)
Ho1-O2	2.293(4)	Ho1-O2	2.323(6)	Ho2-O8	2.285(6)
Ho1-O3	2.321(4)	Ho1-O3	2.362(6)	Ho2-O9	2.283(6)
Ho1-O4	2.311(4)	Ho1-O4	2.268(6)	Ho2-O10	2.319(6)
Ho1-O5	2.328(4)	Ho1-O5	2.320(6)	Ho2-O11	2.337(6)
Ho1-O6	2.313(4)	Ho1-O6	2.318(6)	Ho2-O12	2.341(6)
Ho1-N1	2.515(5)	Ho1-N1	2.495(7)	Ho2-N3	2.511(7)
Ho1-N2	2.518(4)	Ho1-N2	2.530(7)	Ho2-N4	2.516(7)
O1-Ho1-O2	71.77(13)	O1-Ho1-O2	73.8(2)	O7-Ho2-O8	72.1(2)
O3-Ho1-O4	72.95(12)	O3-Ho1-O4	71.5(2)	O9-Ho2-O10	73.5(2)
O5-Ho1-O6	72.37(13)	O5-Ho1-O6	72.7(2)	O11-Ho2-O12	72.0(2)
N1-Ho1-N2	64.38(16)	N1-Ho1-N2	64.5(3)	N3-Ho2-N4	64.6(2)

## Data Availability

Data is contained within the article or supplementary material.

## Supplementary Materials

Non-covalent interactions (ring···ring, C-H(F)···ring, hydrogen bonds) are summarized in Tables S1–S5 for compounds **2**–**6**, respectively. PXRD pattern (Figure S1a,b, S2–S6), packing views (Figures S7–S11) for compounds **2**–**6**, excitation and emission spectra of compounds **2**, **4**, and **5**, recorded in the solid state at room temperature, are given in Figures S12–S14, respectively. CCDC deposition numbers: CCDC 2120112-2120116 contain the supplementary crystallographic data for **2**–**6**, respectively. These data can be obtained free of charge from The Cambridge Crystallographic Data Centre via www.ccdc.cam.ac.uk/data_request/cif.

## Sample Availability

Samples of the compounds are not available.
