# Supplementary material for: Magnetic and Luminescence Properties of 8-Coordinate Holmium(III) Complexes Containing 4,4,4-Trifluoro-1-Phenyl- and 1-(Naphthalen-2-yl)-1,3-Butanedionates
Source: Molecules. 2022 Feb 8;27(3):1129. doi: 10.3390/molecules27031129 (PMC8840565; doi:10.3390/molecules27031129)
Supplement: Supplementary file 1 [file molecules-27-01129-s001.zip › molecules-1553936-supplementary.pdf]

SUPPLEMENTARY MATERIAL

Magnetic and Luminescence Properties of 8-Coordinate Holmium(III) Complexes Containing 4,4,4-Trifluoro-1-phenyl- and 1-(naphthalen-2-yl)-1,3-butanedionates

Franz A. Mautner[1,*], Florian Bierbaumer[1], Ramon Vicente[2], Saskia Speed[2], Annia Tubau[2], Mercè Font-Bardía[3], Roland Fischer[4], Salah S. Massoud[5,6,*]

[1] *Institut für Physikalische und Theoretische Chemie, Technische Universität Graz, Stremayrgasse 9, A-8010 Graz, Austria*
[2] *Departament de Química Inorgànica i Orgànica, Universitat de Barcelona, Martí i Franquès 1-11, E-08028 Barcelona, Spain*
[3] *Departament de Mineralogia, Cristallografia i Dipòsits Minerals and Unitat de Difracció de R-X. Centre Científic i Tecnològic de la Universitat de Barcelona (CCiTUB). Universitat de Barcelona. Solé i Sabarís 1-3. 08028 Barcelona, Spain*
[4] *Institut für Anorganische Chemie, Technische Universität Graz, Stremayrgasse 9, A-8010 Graz, Austria*
[5] *Department of Chemistry, University of Louisiana at Lafayette, P.O. Box 43700, Lafayette, LA 70504, U.S.A.*
[6] *Department of Chemistry, Faculty of Sciences, Alexandria University, Moharam Bey 21511, Alexandria, Egypt*
* Correspondence: E-mail:mautner@tugraz.at, Tel.: +43-316-873-32270, Fax: +43-316-873-8225 (F.A.M.); E-mail: ssmassoud@louisiana.edu, Tel. +01 337-482-5672, Fax: +01 337-482-5676 (S.S. Massoud)

Contents

Title **page #**
Figsures S1a,b, S2-S6: Observed (red) and simulated (blue) PXRD of **1a,b, 2-6**.................... 2
Figsure S7-S11: Packing views of **2-6** 5
Figs. S12-S14: Luminescence excitation and emission spectra of **2**, **4** and **5**. ... 8
Tables S1-S5: Non-coordinative interactions of **2-6** 9

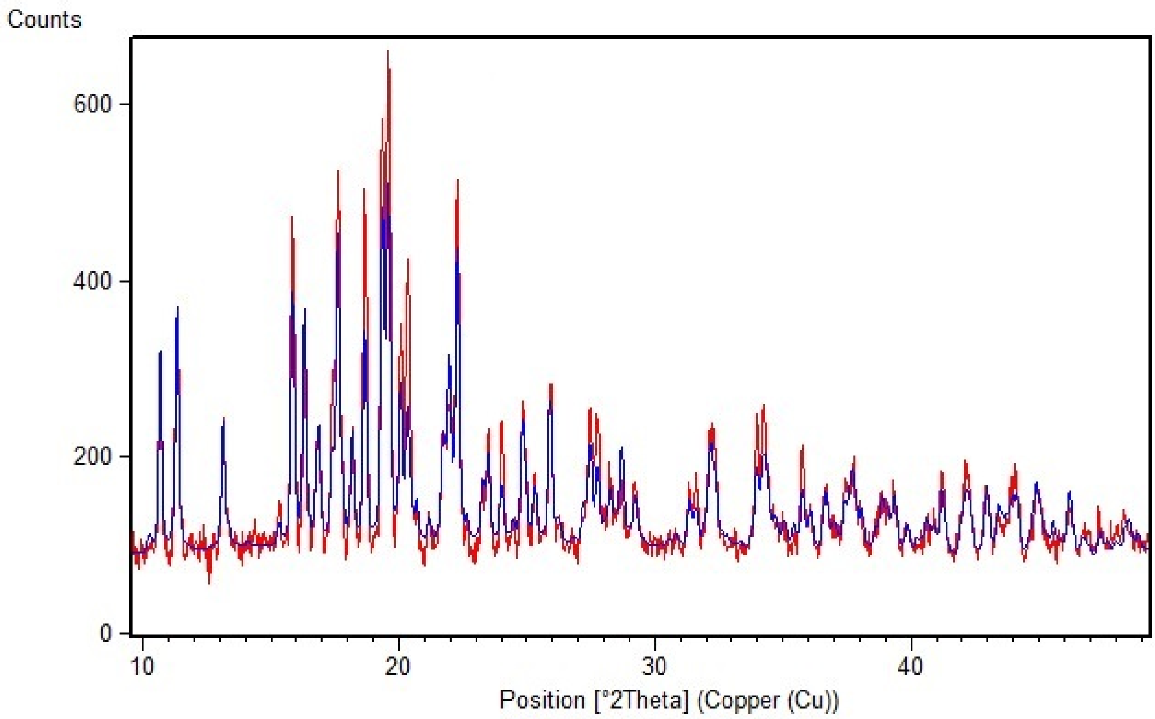

Figure S1a: Observed (red) and simulated (blue) PXRD of **1a**

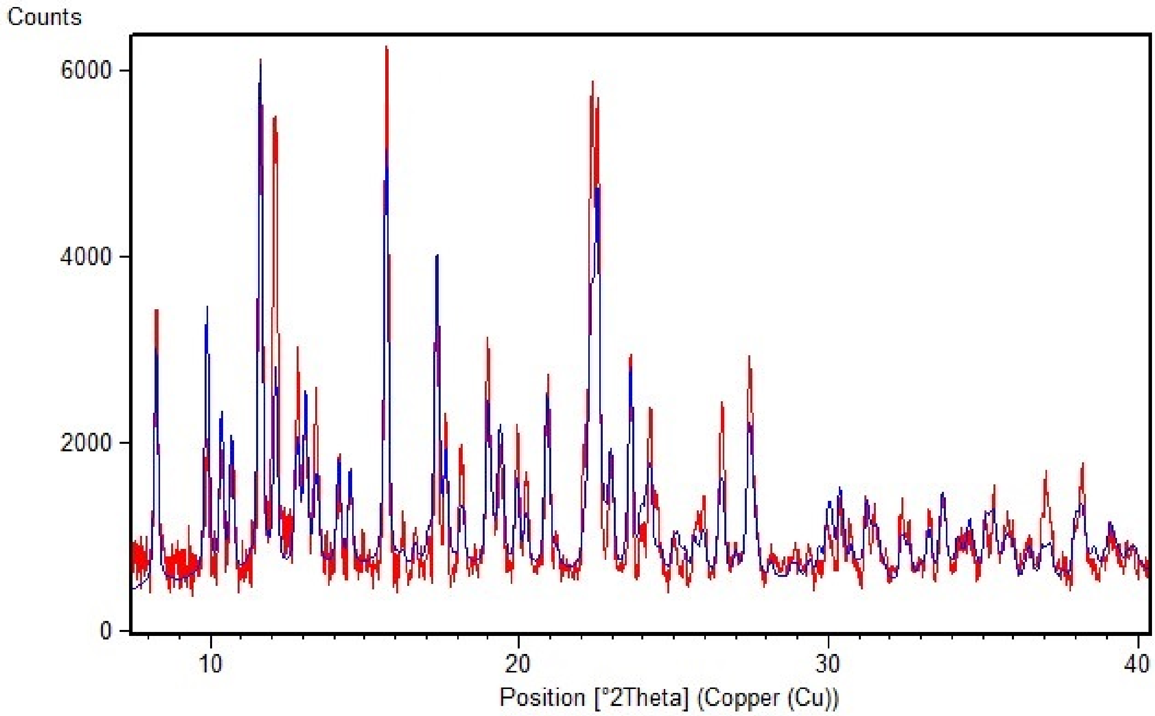

Figure S1b: Observed (red) and simulated (blue) PXRD of **1b**

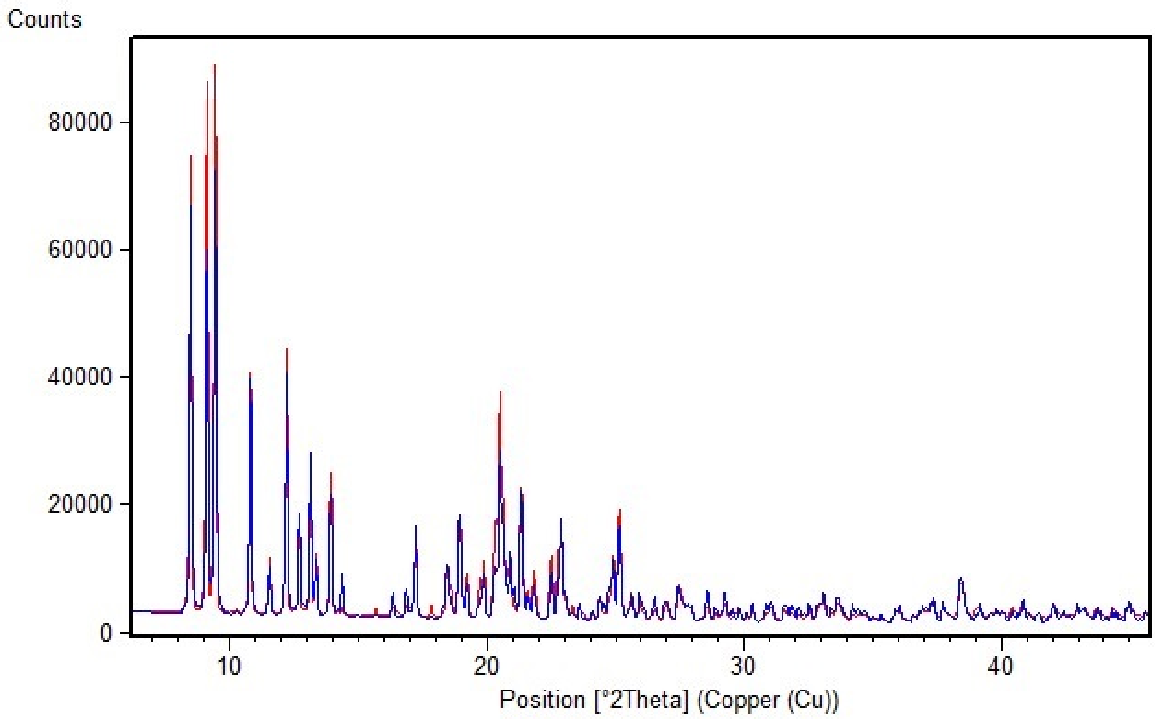

Figure S2: Observed (red) and simulated (blue) PXRD of **2**.

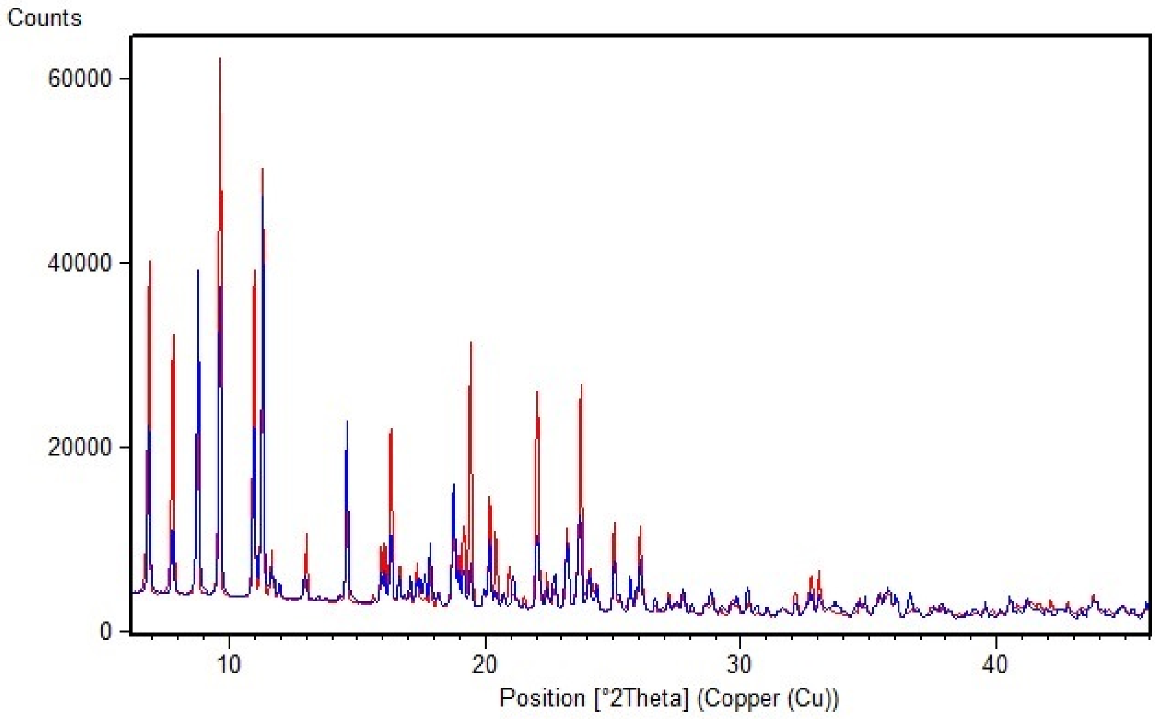

Figure S3: Observed (red) and simulated (blue) PXRD of **3**.

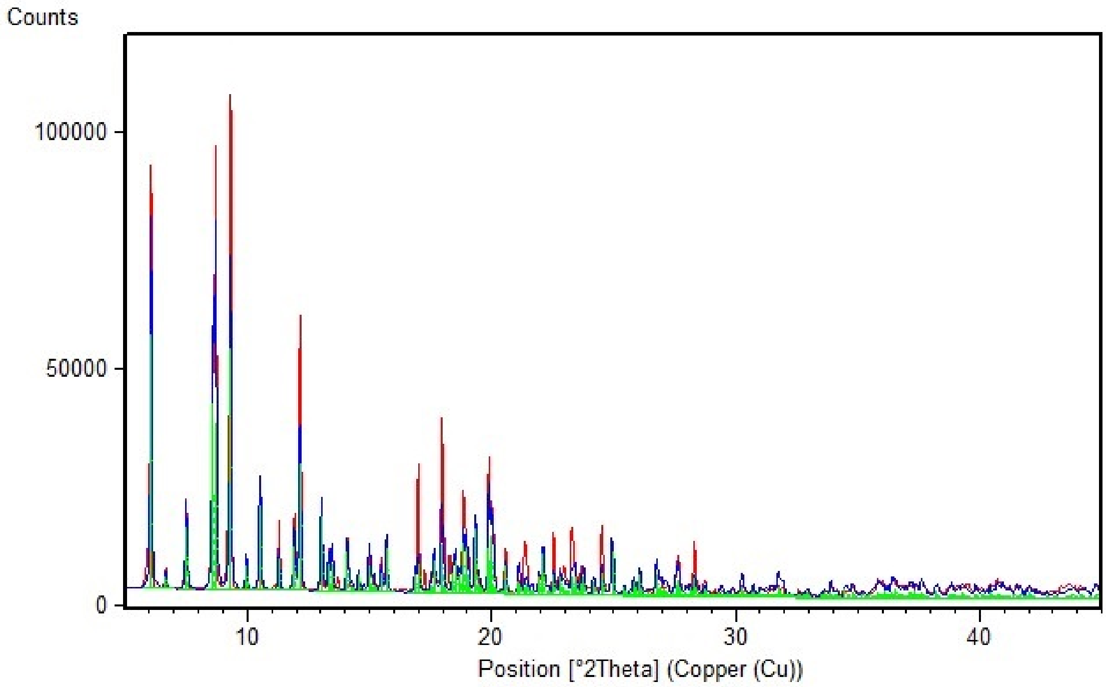

Figure S4: Observed (red) and simulated (blue) PXRD of **4**.

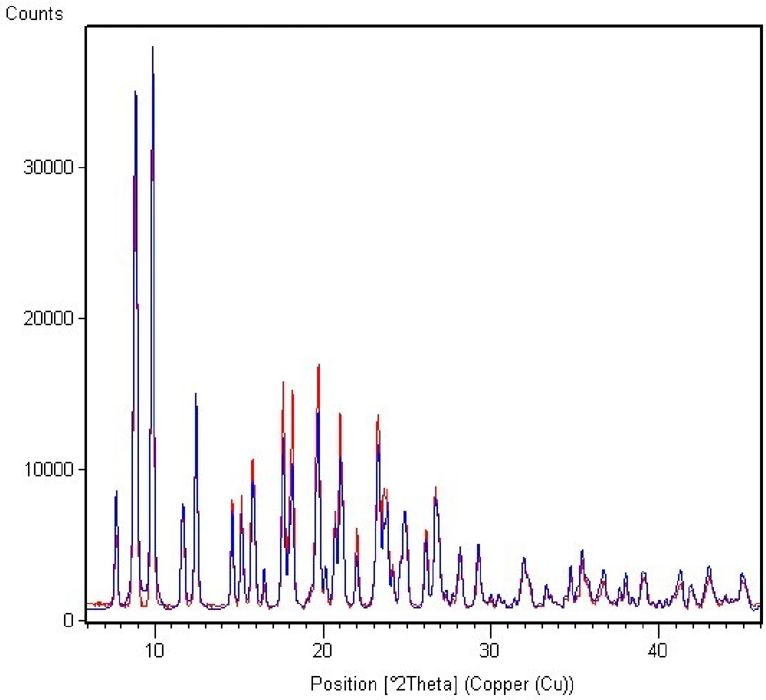

Figure S5: Observed (red) and simulated (blue) PXRD of **5**.

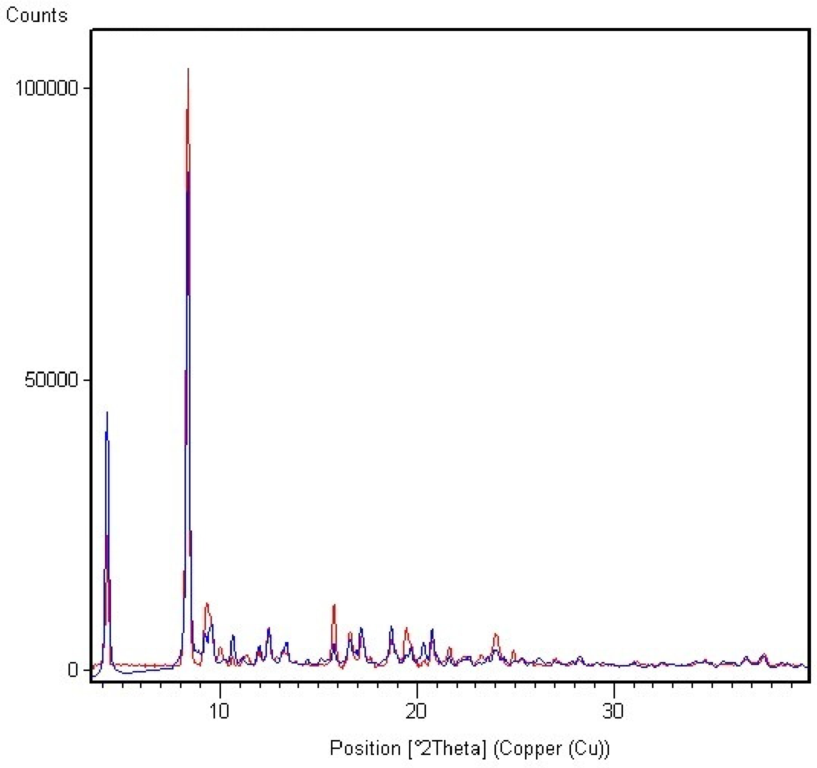

Figure S6: Observed (red) and simulated (blue) PXRD of **6**.

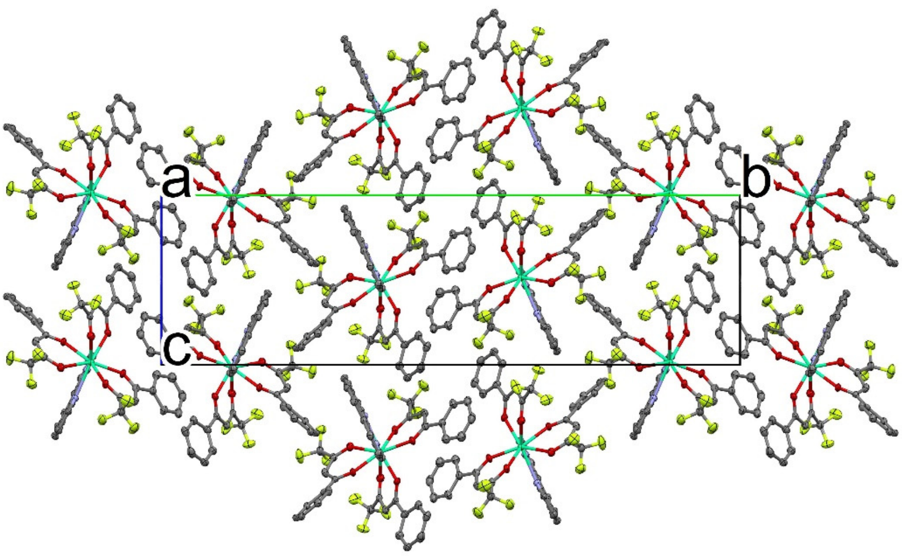

Figure S7: Packing view of **2**.

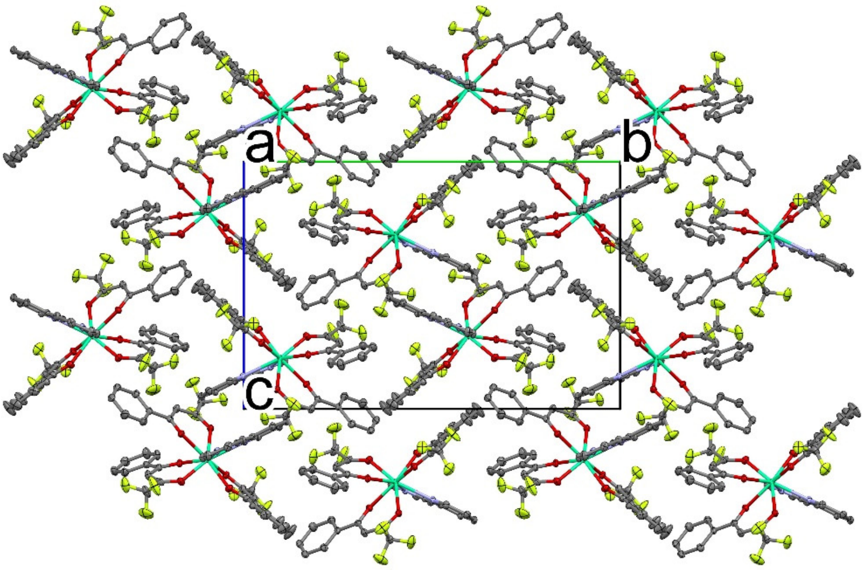

Figure S8: Packing view of **3**.

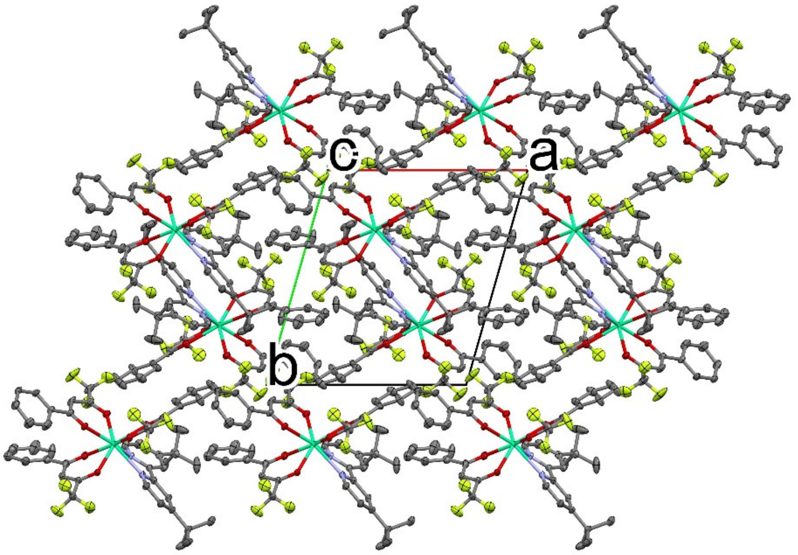

Figure S9: Packing view of **4**.

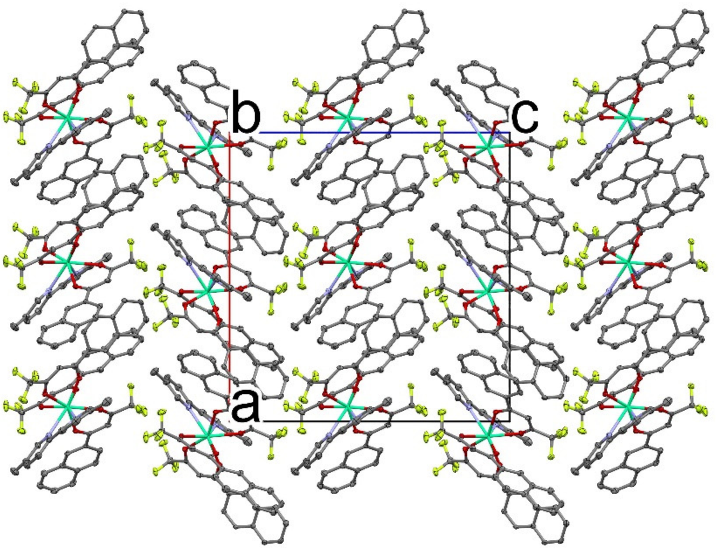

Figure S10: Packing view of **5**.

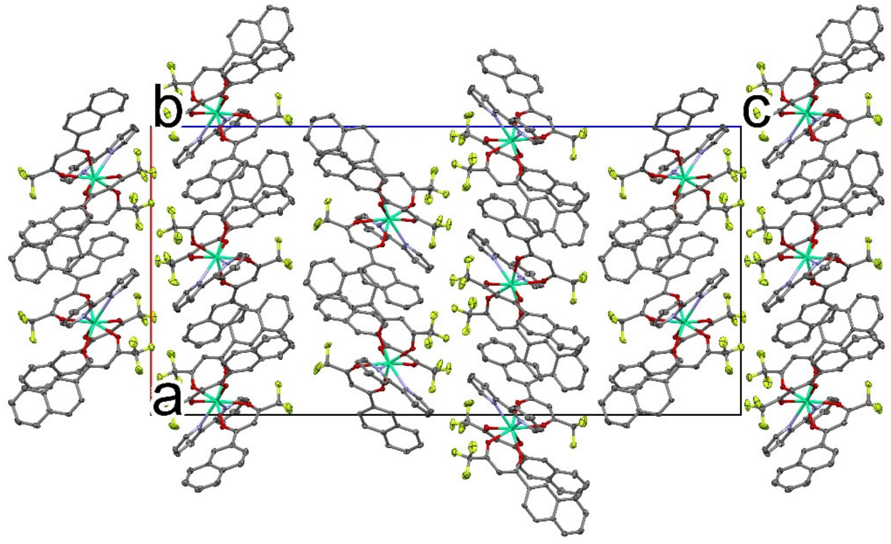

Figure S11: Packing view of **6**.

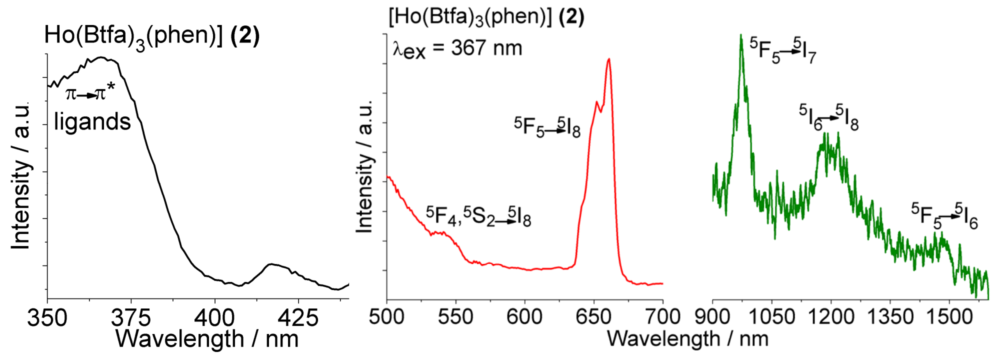

Figure S12. Luminescence excitation (black line), emission in the Visible range (red line) and emission in the NIR range (green line) spectra for compound **2**.

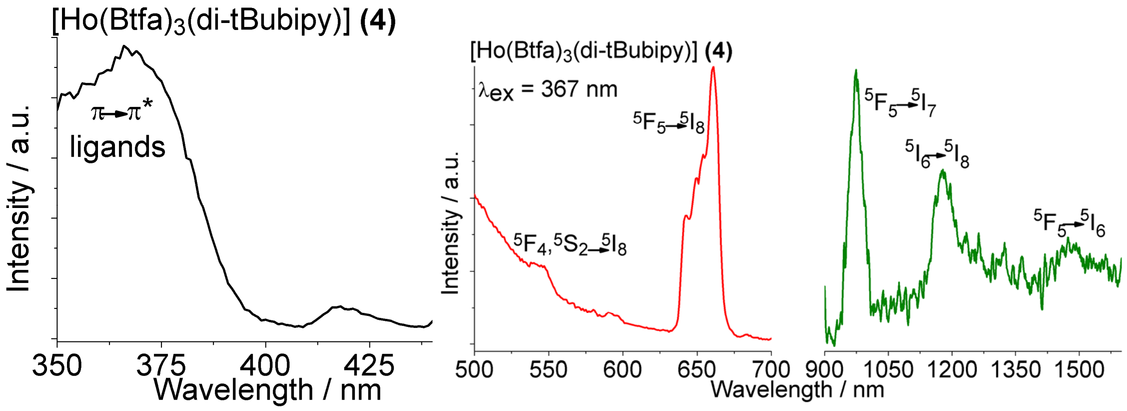

Figure S13. Luminescence excitation (black line), emission in the Visible range (red line) and emission in the NIR range (green line) spectra for compound **4.**

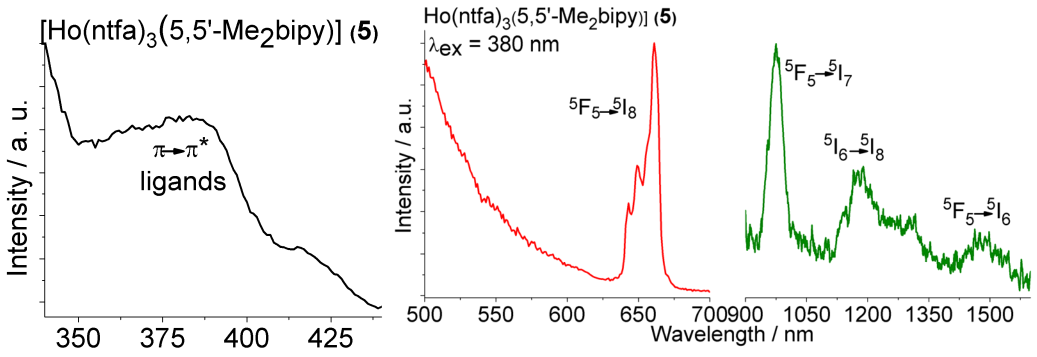

Figure S14. Luminescence excitation (black line), emission in the Visible range (red line) and emission in the NIR range (green line) spectra for compound **5.**

Table S1. Non-coordinative interactions of **2**.

```
Analysis of Ring-Ring Interactions
-----------------------------------
6-Membered Ring ( 1)   N1    -->  C31   -->  C32   -->  C33   -->  C34   -->  C42 (phen)
--------------------------------------------------------------------------------
6-Membered Ring ( 2)   N2    -->  C40   -->  C39   -->  C38   -->  C37   -->  C41 (phen)
--------------------------------------------------------------------------------
6-Membered Ring ( 4)   C15   -->  C16   -->  C17   -->  C18   -->  C19   -->  C20  (benzene)
--------------------------------------------------------------------------------
6-Membered Ring ( 5)   C25   -->  C26   -->  C27   -->  C28   -->  C29   -->  C30 (benzene)
--------------------------------------------------------------------------------
14-Membered Ring ( 9) N1->C31->C32->C33->C34->C35->C36->C37->C38->C39->C40->N2->C41->C42 (phen)
----------------------------------------------------------------------

Cg(I) = Plane number I (= ring number in () above); Cg-Cg = Distance between ring Centroids (Ang.)

Cg(I) Cg(J)[ARU(J)]   Cg-Cg

Cg1 -> Cg5 [4565]  3.967(4) (phen---benzene)
Cg5 -> Cg9 [4564]  3.977(3) (benzene---phen)

[1556] = X,Y,1+Z
[4565] = X,3/2-Y,1/2+Z
[1555] = X,Y,Z
[3666] = 1-X,1-Y,1-Z
[1554] = X,Y,-1+Z
[4564] = X,3/2-Y,-1/2+Z

Analysis of X-H(F)...Cg(Ring) Interactions

Cg(J)   = Center of gravity of ring J (Plane number above); X..Cg = Distance of X to Cg (Angstrom)
H-Perp  = Perpendicular distance of H to ring plane J; X-H(F)..Cg = X-H(F)-Cg angle (degrees)

X--H(I)  Cg(J) [ARU(J)] H(F)..Cg    X-H(F)..Cg     X..Cg

C20 -H20->Cg1  [1554]   2.75  -     138            3.512(8)
C33 -H33->Cg5  [1556]   2.54  -     168            3.474(8)
C1  -F1-> Cg2  [1455]   3.767(6)    115.0(4)       4.496(9)
C11 -F6-> Cg4  [1455]   3.658(6)    162.4(5)       4.930(8)

[1554] = X,Y,-1+Z
[1556] = X,Y,1+Z
[1455] = -1+X,Y,Z

Cg(I) refer to Ring Centre-of-Gravity numbers given in () in Ring-Analysis above

Cg(I)          x               y               z

Cg1         0.5605(3)      0.66637(7)       0.8085(3)
Cg2         0.8514(3)      0.63439(7)       0.5585(3)
Cg4         0.7251(4)      0.56528(8)       0.0489(3)
Cg5         0.6868(3)      0.73885(8)       0.1757(3)
Cg9         0.7329(2)      0.65352(5)     0.70394(18)

Analysis of Potential Hydrogen Bonds

Donor--H..Acceptor  [ARU  ]    D - H    H...A  D...A  D -  H...A

C3  --H3     ..F3   [     ]     0.95     2.33   2.707(9)     103
C13 --H13    ..F5   [     ]     0.95     2.42   2.764(9)     101
C23 --H23    ..F8   [     ]     0.95     2.39   2.741(9)     101
C27 --H27    ..O6   [4564]      0.95     2.60   3.540(9)     171
C28 --H28    ..F7   [4664]      0.95     2.47   3.390(9)     164
C38 --H38    ..O1   [1655]      0.95     2.52   3.237(9)     132
C38 --H38    ..O5   [1655]      0.95     2.59   3.393(9)     143
C40 --H40    ..O4   [     ]     0.95     2.43   3.094(9)     127
Translation of ARU-Code to CIF and Equivalent Position Code

[4564] = [4_575] = x,3/2-y,-1/2+z
[1655] = [1_655] = 1+x,y,z
[4664] = [4_675] = 1+x,3/2-y,-1/2+z
```

Table S2. Non-coordinative interactions of **3**.

Analysis of Ring-Ring Interactions

```
-----------------------------------
  6-Membered Ring ( 1)   N1  -->   C31 -->   C32 -->   C33 -->   C34 -->   C35 (bipy)
-------------------------------------------------------------------------------------
  6-Membered Ring ( 2)   N2  -->   C36 -->   C37 -->   C38 -->   C39 -->   C40 (bipy)
-------------------------------------------------------------------------------------
  6-Membered Ring ( 4)   C15 -->   C16 -->   C17 -->   C18 -->   C19 -->   C20 (benzene)
-------------------------------------------------------------------------------------
  6-Membered Ring ( 5)   C25 -->   C26 -->   C27 -->   C28 -->   C29 -->   C30 (benzene)
---------------------------------------------------------------------------------------------

Cg(I) = Plane number I (= ring number in () above); Cg-Cg = Distance between ring Centroids (Ang.)

Cg(I)    Cg(J)  [ARU(J)]   Cg-Cg

Cg1   -> Cg2    [3666]  3.8412(17)   (bipy---bipy)
Cg4   -> Cg4    [3665]    4.087(2)   (benzene—benzene)
Cg4   -> Cg5    [2555]    4.102(2)   (benzene—benzene)

[3666] = 1-X,1-Y,1-Z
[3665] = 1-X,1-Y,-Z
[2555] = 1/2-X,1/2+Y,1/2-Z

The Cg(I) refer to Ring Centre-of-Gravity numbers given in () in the Ring-Analysis above

Cg(I)           x               y               z

Cg1        0.62812(12)      0.44336(6)      0.33841(8)
Cg2        0.36322(12)      0.55732(6)      0.40843(8)
Cg4        0.44718(14)      0.57478(8)     0.05710(11)
Cg5       -0.09645(13)      0.21271(6)      0.28183(9)
```

Analysis of Potential Hydrogen Bonds

```
Donor --- H....Acceptor [ARU]      D - H      H...A   D...A    D - H...A

C3    --H3     ..F2    [     ]       0.95       2.44   2.763(4)        100
C10   --H10    ..O2    [     ]       0.95       2.42   2.740(4)        1
C13   --H13    ..F6    [     ]       0.95       2.39   2.744(4)        102
C23   --H23    ..F9    [     ]       0.95       2.35   2.719(4)        102
C26   --H26    ..F1    [     ]       0.95       2.55   3.343(4)        141
C31   --H31    ..F7    [     ]       0.95       2.47   3.392(4)        163
C31   --H31    ..O5    [     ]       0.95       2.49   3.130(4)        124
C39   --H39    ..F1    [3566]       0.95       2.46   3.311(4)        149

[3566] = [3_566] = -x,1-y,1-z
```

Table S3. Non-coordinative interactions of **4**.

Analysis of Ring-Ring Interactions and Potential Hydrogen bonds.–No Ring-Ring interactions observed !

Donor	--H....	Acceptor	[ARU]	D - H	H...A	D...A	D - H...A
C3	--H3	..F3	[ ]	0.95	2.38	2.746(7)	103
C13	--H13	..F5	[ ]	0.95	2.45	2.775(7)	100
C20	--H20	..O4	[ ]	0.95	2.41	2.730(8)	100
C23	--H23	..F9	[ ]	0.95	2.36	2.728(6)	103
C28	--H28	..F1	[2656]	0.95	2.51	3.428(8)	162
C41	--H41	..O1	[ ]	0.95	2.53	3.011(6)	112
C43	--H43A	..F3	[1665]	0.98	2.48	3.367(9)	151
C45	--H45A	..F6	[2665]	0.98	2.53	3.456(8)	157

Translation of ARU-Code to CIF and Equivalent Position Code

[2656] = [2_656] = 1-x,-y,1-z
[2665] = [2_665] = 1-x,1-y,-z
[1665] = [1_665] = 1+x,1+y,z

Table S4. Non-coordinative interactions of **5**.

```
Analysis of Ring-Ring Interactions
-----------------------------------
6-Membered Ring ( 1)   N1  --> C43  --> C44  --> C45  --> C46  --> C47 (Me2bipy)
-------------------------------------------------------------------------------
6-Membered Ring ( 3)   C5  --> C6   --> C7   --> C12  --> C13  --> C14
----------------------------------------------------------------------
6-Membered Ring ( 4)   C7  --> C8   --> C9   --> C10  --> C11  --> C12
----------------------------------------------------------------------
6-Membered Ring ( 5)   C19 --> C20  --> C21  --> C26  --> C27  --> C28
----------------------------------------------------------------------
6-Membered Ring ( 7)   C33 --> C34  --> C35  --> C40  --> C41  --> C42
----------------------------------------------------------------------
10-Membered Ring ( 9) C5  ->C6  ->C7  ->C8  ->C9  ->C10 ->C11 ->C12 ->C13 ->C14 (naphthalene A)
------------------------------------------------------------------------------------------
10-Membered Ring (10) C19 ->C20 ->C21 ->C22 ->C23 ->C24 ->C25 ->C26 ->C27 ->C28 (naphthalene B)
------------------------------------------------------------------------------------------
10-Membered Ring (11) C33 ->C34 ->C35 ->C36 ->C37 ->C38 ->C39 ->C40 ->C41 ->C42 (naphthalene C)
------------------------------------------------------------------------------------------

Cg(I) = Plane number I (= ring number in () above); Cg-Cg = Distance between ring Centroids (Ang.)

Cg(I) Cg(J)  [ARU(J)]   Cg-Cg

Cg5  -> Cg7   [4465]    3.614(3) (naphthalene B—Naphthalene C)
Cg5  -> Cg8   [4465]    3.850(3) (naphthalene B—Naphthalene C)
Cg5  -> Cg11  [4465]    3.527(3) (naphthalene B—Naphthalene C)
Cg7  -> Cg10  [4565]    3.520(3) (naphthalene C—Naphthalene B)
Cg10 -> Cg11  [4465]    3.805(3) (naphthalene B—Naphthalene C)

[4465] = -1/2+X,1-Y,Z
[4565] = 1/2+X,1-Y,Z

Analysis of X-H(F)...Cg(Ring) Interactions

Cg(J)   = Center of gravity of ring J (Plane number above); X..Cg = Distance of X to Cg (Angstrom)
H-Perp  = Perpendicular distance of H to ring plane J; X-H(F)..Cg = X-H(F)-Cg angle (degrees)

X--H(I)  Cg(J)  [ARU(J)]  H(F)..Cg    X-H(F)..Cg     X..Cg

C39 -H39 -> Cg3  [4565]                 147          3.734(6)
C51 -H51 -> Cg7  [1565]                 116          3.383(8)
C54 -H54A-> Cg5  [1565]                 117          3.530(8)
C1  -F2  -> Cg4  [3554]    3.026(4)    123.5(3)      3.930(6)
C1  -F2  -> Cg9  [3554]    3.382(4)    140.6(3)      4.502(6)
C29 -F7  -> Cg1  [2675]    3.520(4)    136.3(3)      4.581(6)

[4565] = 1/2+X,1-Y,Z         [1565] = X,1+Y,Z
[3554] = 1/2-X,Y,-1/2+Z      [2675] = 1-X,2-Y,1/2+Z

The Cg(I) refer to Ring Centre-of-Gravity numbers given in () in Ring-Analysis

Cg(I)           x               y               z

Cg1        0.60027(12)       0.9548(2)     0.31663(12)
Cg3        0.19448(12)     0.98730(18)     0.54165(12)
Cg4        0.14285(12)     0.90755(19)     0.64372(12)
Cg5        0.29302(12)     0.44840(18)     0.51408(12)
Cg7        0.62682(12)     0.46228(19)     0.47217(13)
Cg9         0.16861(9)     0.94699(15)      0.59251(9)
Cg10        0.26226(9)     0.43545(15)      0.56700(9)
Cg11        0.66140(9)     0.41067(15)     0.43140(10)

Analysis of Potential Hydrogen Bonds

Donor --H....Acceptor   D - H    H...A  D...A     D - H...A
---------------------------------------------------------------
C3    --H3     ..F1     0.95     2.43   2.753(7)        100
C17   --H17    ..F5     0.95     2.35   2.720(7)        103
C31   --H31    ..F9     0.95     2.40   2.760(7)        102
C34   --H34    ..O6     0.95     2.36   2.712(6)        101
C47   --H47    ..O3     0.95     2.50   3.045(7)        117
```

Table S5. Non-coordinative interactions of **6**.

```
Analysis of Ring-Ring Interactions
-----------------------------------
6-Membered Ring ( 3)   C5    -->   C6   -->    C7    -->   C12   -->   C13  -->   C14
-------------------------------------------------------------------------------------
6-Membered Ring ( 5)   C19   -->   C20   -->   C21   -->   C26   -->   C27   -->   C28
-------------------------------------------------------------------------------------
6-Membered Ring ( 7)   C33   -->   C34   -->   C35   -->   C40   -->   C41   -->   C42
-------------------------------------------------------------------------------------
6-Membered Ring ( 8)   C35   -->   C36   -->   C37   -->   C38   -->   C39   -->   C40
-------------------------------------------------------------------------------------
10-Membered Ring ( 9)  C5 --> C6 --> C7 --> C8 --> C9 -->C10 -->C11-->C12-->C13 -->C14
-------------------------------------------------------------------------------------
10-Membered Ring (10)  C19 -->C20 -->C21 -->C22 -->C23 -->C24 -->C25-->C26-->C27-->C28
-------------------------------------------------------------------------------------
10-Membered Ring (11)  C33 -->C34 -->C35 -->C36 -->C37 -->C38 -->C39-->C40-->C41-->C42
-------------------------------------------------------------------------------------
6-Membered Ring (13)   N4    -->   C100  -->   C101  -->   C102  -->   C103  -->  C104 (bipy)
-------------------------------------------------------------------------------------
6-Membered Ring (14)   C57   -->   C58   -->   C59   -->   C64   -->   C65   -->   C66
-------------------------------------------------------------------------------------
6-Membered Ring (18)   C85   -->   C86   -->   C87   -->   C92   -->   C93   -->   C94
-------------------------------------------------------------------------------------
6-Membered Ring (19)   C87   -->   C88   -->   C89   -->   C90   -->   C91   -->   C92
-------------------------------------------------------------------------------------
10-Membered Ring (20)  C57 -->C58 -->C59 -->C60 -->C61 -->C62 -->C63-->C64-->C65-->C66
-------------------------------------------------------------------------------------
10-Membered Ring (21)  C71 -->C72 -->C73 -->C74 -->C75 -->C76 -->C77-->C78-->C79-->C80
-------------------------------------------------------------------------------------
10-Membered Ring (22)  C85 -->C86 -->C87 -->C88 -->C89 -->C9 -->C91-->C92 -->C93-->C94
-------------------------------------------------------------------------------------
HINT: 10-membered rings (9, 10, 11, 20, 21, 22 refer to naphthalene rings A,B,C,D,E,F, resp.)
--------------------------------------------------------------------------------------------

Cg(I) = Plane number I (= ring number in () above); Cg-Cg = Distance between ring Centroids (Ang.)

Cg(I) Cg(J)  [ARU(J)]   Cg-Cg

Cg3  -> Cg7  [4545]    3.659(5)
Cg3  -> Cg8  [4545]    3.935(5)
Cg3  -> Cg11 [4545]    3.597(5)
Cg7  -> Cg9  [4445]    3.767(5)
Cg16 -> Cg18 [4465]    3.643(5)
Cg16 -> Cg19 [4465]    3.893(5)
Cg16 -> Cg22 [4465]    3.563(4)
Cg18 -> Cg21 [4565]    3.710(5)

[4465] = -1/2+X,3/2-Y,Z       [4545] = 1/2+X,-1/2-Y,Z
[4565] = 1/2+X,3/2-Y,Z        [4445] = -1/2+X,-1/2-Y,Z
```

Analysis of X-H(F)...Cg(Ring) Interactions

```
Cg(J)   = Center of gravity of ring J (Plane number above); X..Cg = Distance of X to Cg (Angstrom)
H-Perp  = Perpendicular distance of H to ring plane J; X-H(F)..Cg = X-H(F)-Cg angle (degrees)

X--H(I)  Cg(J)    [ARU(J)]  H(F)..Cg   X-H(F)..Cg     X..Cg

C11  -H11 -> Cg5   [1545]   2.85        163          3.767(11)
C44  -H44 -> Cg7   [1565]   2.79        126          3.437(10)
C44  -H44 -> Cg11  [1565]   2.89        140          3.667(11)
C46  -H46 -> Cg3   [1565]   2.86        112          3.343(10)
C49  -H49 -> Cg8   [1565]   2.88        132          3.591(12)
C66  -H66 -> Cg13  [4455]   2.85        137          3.602(9)
C77  -H77 -> Cg14  [1565]   2.52        152          3.394(10)
C77  -H77 -> Cg20  [1565]   2.73        139          3.510(10)
C96  -H96 -> Cg18  [1545]   2.xx        129          3.465(9)
C96  -H96 -> Cg22  [1545]   2.xx        141          3.742(9)
C101-H101 -> Cg19  [1545]   2.86        131          3.553(11)

[1545] = X,-1+Y,Z
[1565] = X,1+Y,Z
[4455] = -1/2+X,1/2-Y,Z
[1565] = X,1+Y,Z
```

```
[1545] = X,-1+Y,Z

Cg(I) refer to Ring Centre-of-Gravity numbers given in () in Ring-Analysis

Cg(I)          x              y              z

Cg3       0.68668(17)      -0.3170(3)      0.65482(8)
Cg5       0.80662(16)       0.2226(3)      0.66122(8)
Cg7       0.35118(18)      -0.2548(3)      0.62918(8)
Cg8         0.2855(2)      -0.3580(4)     0.58943(10)
Cg9       0.71083(16)      -0.3391(3)      0.68042(7)
Cg10      0.82885(14)       0.1763(3)      0.68520(6)
Cg11      0.31826(15)      -0.3064(3)      0.60931(7)
Cg13      0.95363(19)       0.2064(4)      0.45126(9)
Cg14      0.56390(16)       0.2664(3)      0.35022(8)
Cg18      1.01688(17)       0.7607(3)      0.38548(8)
Cg19      1.08335(17)       0.8633(3)      0.42543(9)
Cg20      0.54546(14)       0.2814(3)      0.32306(7)
Cg21      0.65600(15)       0.8533(3)      0.33865(7)
Cg22      1.05013(13)       0.8117(2)      0.40543(7)
```

Analysis of Potential Hydrogen Bonds

```
Donor --H....Acceptor [ARU]    D - H   H...A   D...A  D - H...A
---------------------------------------------------------------
C3    --H3     ..F2   [     ]    0.95    2.39   2.756(12)   102
C8    --H8     ..F18  [3645]    0.95    2.50   3.212(13)   132
C17   --H17    ..F9   [     ]    0.95    2.41   2.757(12)   101
C20   --H20    ..O4   [     ]    0.95    2.33   2.671(10)   100
C27   --H27    ..O6   [4555]    0.95    2.57   3.356(10)   140
C31   --H31    ..F5   [     ]    0.95    2.35   2.730(11)   103
C34   --H34    ..O6   [     ]    0.95    2.38   2.734(10)   102
C36   --H36    ..F14  [4455]    0.95    2.46   3.402(12)   170
C47   --H47    ..O5   [     ]    0.95    2.58   3.029(12)   109
C52   --H52    ..O1   [     ]    0.95    2.58   3.142(12)   118
C55   --H55    ..F11  [     ]    0.95    2.37   2.740(11)   103
C69   --H69    ..F15  [     ]    0.95    2.36   2.717(11)   101
C83   --H83    ..F17  [     ]    0.95    2.34   2.719(12)   103
C86   --H86    ..O12  [     ]    0.95    2.39   2.724(10)   100
C88   --H88    ..F1   [4555]    0.95    2.47   3.398(11)   164
C99   --H99    ..O11  [     ]    0.95    2.59   3.030(10)   109
C104  --H104   ..O9   [     ]    0.95    2.53   3.117(11)   120

No Classic Hydrogen Bonds Found

Translation of ARU-Code to CIF and Equivalent Position Code

[4455] = [3_455] = -1/2+x,1/2-y,z
[3645] = [4_645] = 3/2-x,-1/2+y,1/2+z
[4555] = [3_555] = 1/2+x,1/2-y,z
```
